# The Effect of Oil-Based Cannabis Extracts on Metabolic Parameters and Microbiota Composition of Mice Fed a Standard and a High-Fat Diet

**DOI:** 10.3390/ijms25021073

**Published:** 2024-01-16

**Authors:** Adi Eitan, Ofer Gover, Liron Sulimani, David Meiri, Naama Shterzer, Erez Mills, Betty Schwartz

**Affiliations:** 1 The Institute of Biochemistry, Food Science and Nutrition, The Robert H. Smith Faculty of Agriculture, Food and Environment, The Hebrew University of Jerusalem, Rehovot 9190401, Israel; adi.levy@mail.huji.ac.il (A.E.); ofer.gover@mail.huji.ac.il (O.G.); 2 Cannasoul Analytics, 9 Tarshish Industrial Park, Caesarea 3079822, Israel; liron@cannasoul.co.il; 3 The Laboratory of Cancer Biology and Cannabinoid Research, Department of Biology, Technion-Israel Institute of Technology, Haifa 3200003, Israel; dmeiri@technion.ac.il; 4 The Department of Animal Sciences, The Robert H. Smith Faculty of Agriculture, Food and Environment, The Hebrew University of Jerusalem, Rehovot 9190401, Israelerez.mills@mail.huji.ac.il (E.M.)

**Keywords:** obesity, cannabis, endocannabinoid system, microbiota

## Abstract

The prevalence of obesity and obesity-related pathologies is lower in frequent cannabis users compared to non-users. It is well established that the endocannabinoid system has an important role in the development of obesity. We recently demonstrated that prolonged oral consumption of purified Δ-9 Tetrahydrocannabinol (THC), but not of cannabidiol (CBD), ameliorates diet-induced obesity and improves obesity-related metabolic complications in a high-fat diet mouse model. However, the effect of commercially available medical cannabis oils that contain numerous additional active molecules has not been examined. We tested herein the effects of THC- and CBD-enriched medical cannabis oils on obesity parameters and the gut microbiota composition of C57BL/6 male mice fed with either a high-fat or standard diet. We also assessed the levels of prominent endocannabinoids and endocannabinoid-like lipid mediators in the liver. THC-enriched extract prevented weight gain by a high-fat diet and attenuated diet-induced liver steatosis concomitantly with reduced levels of the lipid mediators palmitoyl ethanolamide (PEA) and docosahexaenoyl ethanolamide (DHEA) in the liver. In contrast, CBD-enriched extract had no effect on weight gain, but, on the contrary, it even exacerbated liver steatosis. An analysis of the gut microbiota revealed that mainly time but not treatment exerted a strong effect on gut microbiota alterations. From our data, we conclude that THC-enriched cannabis oil where THC is the main constituent exerts the optimal anti-obesity effects.

## 1. Introduction

Obesity is a major risk factor for the development of multiple diseases such as type-2 diabetes, non-alcoholic fatty liver disease (NAFLD), and cardiovascular disorders [1]. It is well established that the endocannabinoid system (ECS) plays a major role in the development of obesity by regulating appetite, metabolic processes, and energy balance [2]. Dysregulation of the ECS results in a high endocannabinoid ‘tone’, leading to increased appetite, lipogenesis, adipogenesis, and a decrease in energy expenditure, which further escalates adiposity, thus creating a vicious cycle [2,3]. The central role of this system in obesity development makes it a worthy therapeutic target for the treatment of obesity and associated comorbidities.

The ECS is a complex system comprised of several bioactive lipids of which the two most-studied are *N*-arachidonoylethanolamide (AEA) and 2-arachidonoylglycerol (2-AG) [4]. These bioactive lipids interact with both membrane-bound and nuclear receptors, leading to a broad range of physiological effects [4]. The two main endocannabinoid receptors are the G-coupled receptors CB1 and CB2, which are expressed throughout the central nervous system and many other tissues, including organs affecting metabolic homeostasis [4]. In recent years, additional receptors and “endocannabinoid-like” mediators have been identified as part of the extended ECS [5]. Among them are congeners of AEA (*N*-acylethanolamines (NAEs)) and 2-AG (2-monoacyleglycerols (2-MAGs)), and *N*-acyl-amino acids (NAAs) [5].

Previous studies have demonstrated the link between the ECS and the gut microbiota in relation to obesity. For example, a blockade of CB1 was suggested to ameliorate diet-induced obesity by increasing the relative abundance of *Akkermansia muciniphila*, a commensal Gram-negative bacterium with beneficial effects on metabolism [6]. Moreover, alterations in the ECS are implicated in the dysregulation of lipopolysaccharide (LPS) levels, gut integrity disruption, and dysbiosis of the gut microflora [7]. Microbiota composition is altered in obese settings, and compelling evidence suggests that these alterations influence several metabolic pathways that contribute to obesity-induced metabolic disorders [8]. Decreased alpha diversity and changes in the ratio of Bacteroidetes to Firmicutes are some of the markers that are associated with obesity [8].

The ECS can be activated by the consumption of exogenous cannabinoids, such as phytocannabinoids found in the *Cannabis sativa* (*C. sativa*) plant [9]. The *C. sativa* plant has been used as an effective therapeutic agent for thousands of years. The plant contains hundreds of natural compounds of which more than 100 have been identified as phytocannabinoids [10]. The major two are cannabidiol (CBD) and Δ9-tetrahydrocannabinol (THC), the latter being the predominant psychoactive component in *C. sativa*. Both demonstrated therapeutic properties in different settings [10,11]. In recent years, there has been an increase in the use of purified cannabinoids and medical cannabis, mainly in the form of whole plant extracts that contain, in addition to phytocannabinoids, a full spectrum of metabolites with potential therapeutic properties, such as terpenes and flavonoids [12,13]. For example, cannflavins, prenylated flavonoids found in *C. sativa,* exert anti-inflammatory activities by inhibiting the microsomal prostaglandin E2 (PGE2) synthase-1 and the 5-lipoxygenase activities, leading to reduced PGE2 and leukotriene production, respectively [14]. Medical cannabis is commonly used as an appetite stimulator in pathological conditions such as cancer and HIV [11]. Paradoxically, several studies have established the association between cannabis exposure and reduced risk of obesity and metabolic diseases [15,16,17,18]. Moreover, various compounds comprising cannabis, as well as whole cannabis extracts, have been shown to exert anti-obesity properties in animal models [19,20,21,22,23]. In this regard, the effect of THC was suggested to be, in part, due to alterations in the gut microbiota [19].

We have recently shown that prolonged oral consumption of purified THC, but not CBD, ameliorates diet-induced obesity and improves obesity-related metabolic complications in a high-fat diet mouse model [24]. In the same experimental model, we investigated the effect of cannabis-based oil preparations enriched with either THC (Erez) or CBD (Avidekel) on obesity-related metabolic parameters, and these are reported herein.

To our knowledge, no studies hitherto have examined the effect of commercially available medical cannabis oil-based preparations on obesity parameters in a rodent model. Based on our recent findings and the data stated above, we hypothesize that prolonged exposure to whole plant extracts may produce anti-obesity properties, possibly through alterations in the gut microbiota. In the current study, we investigate the effect of orally administered THC- and CBD-enriched medical cannabis oils (Erez and Avidekel, respectively, provided by Tikun Olam) in mice fed a normal diet or a high-fat diet inducing obesity, with emphasis on microbiota alterations.

## 2. Results

### 2.1. Erez (THC-Enriched) but Not Avidekel (CBD-Enriched) Reduces Weight Gain

**Figure 1:**
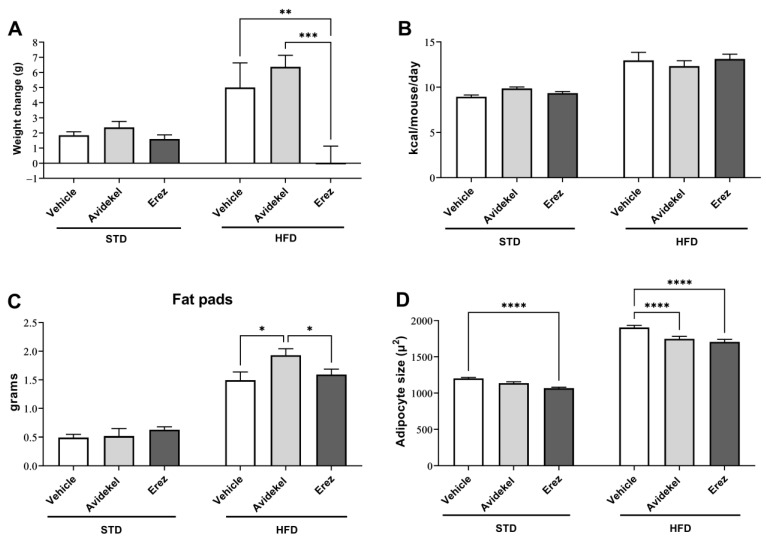
Effect of Avidekel and Erez extracts on weight gain, caloric intake, fat pad weights, and adipocyte size. Body weight change (**A**), average caloric intake (**B**), fat pad weights (**C**), and quantitative analysis of adipocyte size in epididymal tissue (**D**) were measured and calculated at the end of the treatment regime (*n* = 5–7). Data are shown as mean ± SEM. * *p* < 0.05, ** *p* < 0.002, *** *p* < 0.001, **** *p* < 0.0001. Statistical significance was determined by two-way ANOVA followed by Tukey’s post hoc test.

The effect of prolonged oral consumption of CBD-enriched (Avidekel) and THC-enriched (Erez) medical cannabis oils was evaluated in mice fed a standard diet (STD) and a high-fat diet (HFD). The oil preparations used in the study were analyzed for cannabinoid and terpene content (Supplemental Figures S1 and S2 and Tables S2 and S3). Treatment with medical cannabis oils did not affect weight gain, caloric intake, or fat pad weights in mice fed a standard diet, though adipocyte size was decreased in mice treated with Erez extract (Figure 1A–C, left columns). In mice fed a high-fat diet, treatment with Erez extract prevented weight gain, while treatment with Avidekel extract significantly increased fat pad weights (Figure 1A,C). Both treatments decreased adipocytes in the epididymal fat (Figure 1D).

### 2.2. Avidekel Increases Adipose Tissue Inflammation in STD Mice

**Figure 2:**
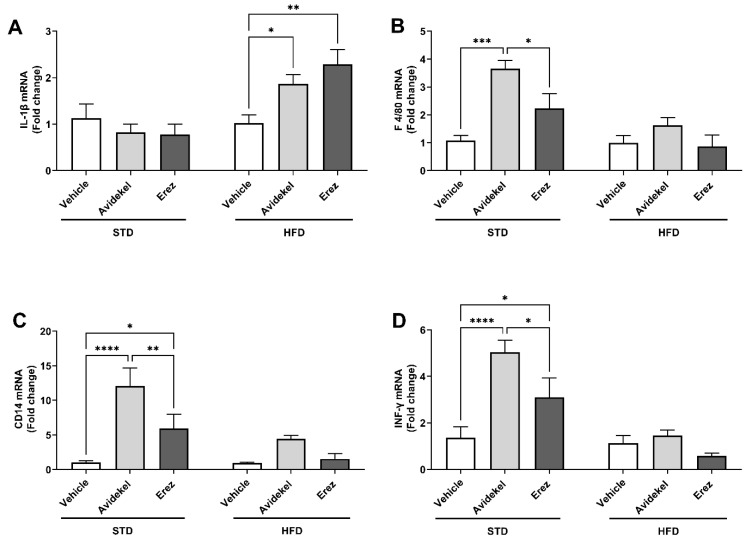
Inflammation markers in adipose tissue. mRNA expression of genes involved in inflammation examined in epididymal fat: (**A**). Interleukin-1 beta (IL-1β). (**B**). Macrophage receptor F4/80. (**C**). Monocyte differentiation antigen CD14. (**D**). Interferon-γ (INF-γ). PPIA was used as a housekeeping gene. The gene expression levels were calculated as ΔΔCT and normalized to the vehicle of each diet (*n* = 4–6). Data are shown as the mean of fold-change ± SEM. * *p* < 0.05, ** *p* < 0.002, *** *p* < 0.001, **** *p* < 0.0001. Statistical significance was determined by two-way ANOVA followed by Tukey’s post hoc test.

Abnormal expansion of the adipose tissue, as seen in obesity, promotes inflammation [25]. Thus, we next examined whether the decreased adiposity and weight gain by using Erez extract were associated with decreased adipose inflammation. In mice fed a high-fat diet, both Erez (*p* < 0.002) and Avidekel (*p* < 0.05) extracts increased the gene expression of the pro-inflammatory cytokine IL-1β (Figure 2A). In mice on a standard diet, Avidekel extract significantly increased adipose tissue inflammation, as demonstrated by the increased gene expression of markers abundant on the surface of the monocytes and macrophages F4/80 (*p* < 0.001, Figure 2B) and CD14 (*p* < 0.0001, Figure 2C), in addition to the pro-inflammatory cytokine INF-γ (*p* < 0.0001, Figure 2D). The last two were also upregulated by using Erez extract compared to vehicle-treated mice (Figure 2C,D). Notably, mice treated with Erez extract exhibited significantly less adipose inflammation compared to those treated with Avidekel (Figure 2B–D).

### 2.3. Erez Extract Improves Diet-Induced Liver Steatosis

**Figure 3:**
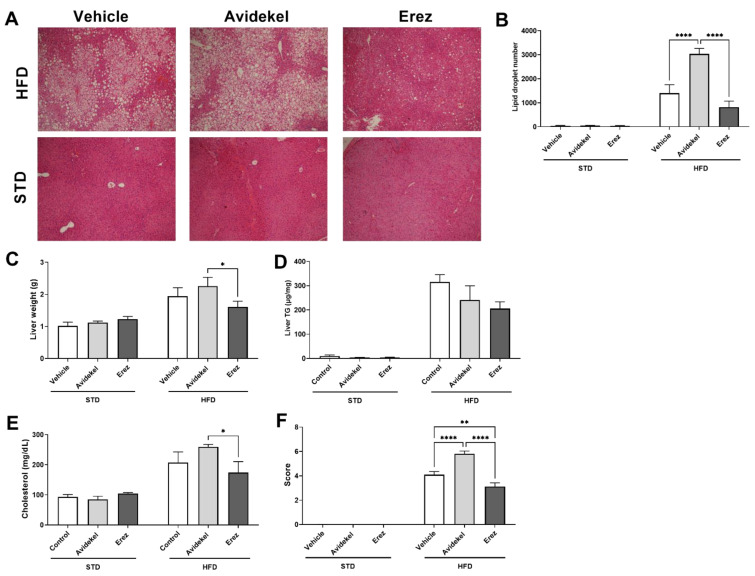
Effect of Avidekel and Erez extract on obesity-induced steatosis. (**A**). Representative liver tissue sections stained with H&E (magnification ×100). (**B**). Morphological analysis of liver droplet density (*n* = 5). (**C**). Liver weights. (**D**). Levels of hepatic triglycerides (*n* = 3). (**E**). Serum cholesterol (*n* = 3). (**F**). Severity of steatosis was blindly scored as described by Liang et al. [27] (*n* = 3). Data are shown as mean ± SEM. * *p* < 0.05, ** *p* < 0.002, **** *p* < 0.0001. Statistical significance was determined by using two-way ANOVA followed by Tukey’s post hoc test.

A central pathology of obesity is fatty liver disease, which is instigated by excessive accumulation of lipids in the liver (steatosis) [26]. Thus, we next examined the effect of Avidekel and Erez extracts on liver parameters. HFD-induced fatty liver, as indicated by increased lipid droplets, liver weight, hepatic triglyceride content, steatosis score, and serum cholesterol (Figure 3A–F). Avidekel treatment further exacerbated liver steatosis, significantly increasing lipid droplets compared to vehicle and Erez treatments (Figure 3B). Notably, Erez treatment alleviated liver steatosis compared to Avidekel treatment, as demonstrated by the decreased liver weight, serum cholesterol levels, and steatosis score (Figure 3C,E,F, respectively). The latter was also significantly lower compared to vehicle-treated mice (Figure 3F).

### 2.4. Erez Extract Reduces Liver Levels of DHEA and PEA in High-Fat Diet-Fed Mice

The endocannabinoid system plays a major role in obesity-induced liver steatosis [28]. To determine whether the improvement in liver steatosis by using Erez extract was mediated by modulation of the ECS, we next examined in mice fed a high-fat diet the levels of the well-known endocannabinoids AEA and 2-AG, as well as other family members of NAEs, 2-MAGs, and NAAs that together act as mediators within the endocannabinoidome [5]. No significant differences were observed in AEA and 2-AG levels in livers from vehicle- or extract-treated mice, though a trend toward lowered 2-AG levels could be seen in mice treated with medicinal cannabis oils. Mice treated with Erez extract demonstrated decreased levels of NAEs (DHEA and PEA), compared to vehicle-treated mice (Table 1).

**Figure 4:**
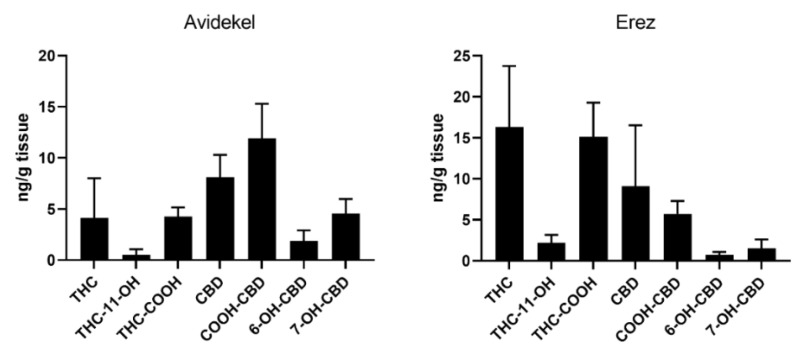
Levels of phytocannabinoid metabolites in liver tissue. Levels of THC, CBD, and their metabolites were assessed at the end of the treatment regime as described in materials and methods. Data are shown as mean ± SEM (*n* = 4).

The presence of THC and CBD and their respective metabolites were assessed in liver tissues to examine the pharmacological exposure to the active molecules. Both THC and CBD and their respective metabolites were present in the liver tissues (Figure 4). As expected, mice treated with Avidekel extract demonstrated elevated levels of CBD compared to THC and vice versa in mice treated with Erez extract (Figure 4).

### 2.5. Effect of Avidekel and Erez Extracts on the Microbiota

Interaction between the ECS and the gut microbiota is suggested to take part in the development of obesity and obesity-related metabolic disorders [6,7,19]. We performed an alpha diversity analysis, which characterizes the microbiota community in three ways: observed richness, evenness, and Shannon diversity index, a metric that weighs the number of species by their relative evenness data [29]. In addition, we looked at the Firmicutes/Bacteroidetes (F/B) ratio, which is associated with maintaining homeostasis [30]. Four different time points were chosen to assess how diet and treatment influenced microbiota composition. T1 represents microbiota after one month of HFD; T2 represents microbiota one day before the start of treatment; T3 represents microbiota one day after the start of treatment; and T4 represents microbiota on the day of sacrifice.

**Figure 5:**
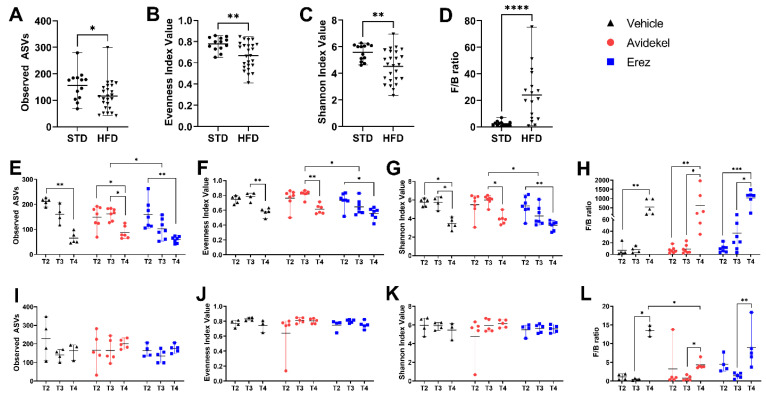
Alpha diversity and F/B ratios of gut microbiota in mice. Fecal samples were taken at various time points (T1—a month after starting HFD; T2—a day before the start of treatment; T3—one day after the start of the treatment; and T4—day of sacrifice), and gut microbiota were analyzed. The gut microbial richness was estimated by observed species; the diversity was evaluated by using the Shannon index; and Evenness was measured by using the Shannon Evenness index. Alpha diversity metrics: richness, evenness, diversity, and Firmicutes/Bacteroidetes ratio at T1 (**A**–**D**), HFD-fed mice (**E**–**H**), and STD-fed mice (**I**–**L**). The line shows the average, and the whiskers show the minimum and maximum (*n* = 4–7). * *p* < 0.05, ** *p* < 0.002, *** *p* < 0.001, **** *p* < 0.0001. Statistical significance was calculated by using non-parametric Mann–Whitney (**A**–**D**) and Kruskal–Wallis (**E**–**L**) tests.

**Figure 6:**
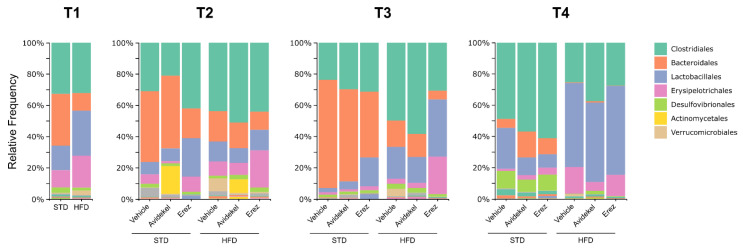
The relative frequency (%) of microbial taxa at the order level in mice gut microbiota samples. Each vertical bar represents the average fecal sample of the respective treatment group at four different time points (T1—a month after starting HFD; T2—a day before the start of treatment; T3—one day after the start of the treatment; and T4—day of sacrifice). Data are shown as percentages (*n* = 4–7).

At T1, the richness, evenness, and diversity of the microbial community (alpha diversity) in mice fed a high-fat diet was lower compared to mice fed a standard diet, whereas the F/B ratio was increased by a high-fat diet (Figure 5A–D). In mice fed a high-fat diet, alterations in gut microbiota could be observed within each group and between groups at different time points after treatment commencement (Figure 5E–H). Considering time as a factor, all treatment groups demonstrated reduced alpha diversity and an increase in the F/B ratio over time (Figure 5E–H). Considering treatment as a factor, at T3, a significant shift in microbiota composition could be seen in mice treated with Erez extract compared to Avidekel, as indicated by the lower alpha diversity metrics of the former (Figure 5E–G). No differences were observed between all treatment groups at T4. Mice fed a standard diet exhibited a higher F/B ratio over time, and when considering treatment as a factor, mice treated with Avidekel extract showed a decreased F/B ratio at T4 compared to vehicle-treated mice (Figure 5L). The gut microbial community of mice fed a high-fat diet, or a standard diet, exhibited a decrease in the relative abundance of bacteroidales at T4, which was accompanied by an increase in Lactobacillales in mice fed a high-fat diet and an increase in clostridiales in mice fed a standard diet (Figure 6).

## 3. Discussion

We previously demonstrated that prolonged oral treatment with THC prevented HFD-induced body weight gain and improved metabolic alterations after diet-induced obesity in C57BL/6 male mice [24]. Here we show that prolonged oral administration of THC-enriched medical cannabis oil (Erez extract) similarly prevented HFD-induced weight gain and improved liver steatosis. These effects were associated with decreased levels of liver PEA and DHEA. In contrast, CBD-enriched medical cannabis oil (Avidekel extract) demonstrated a contrasting effect to that of Erez extract and had no beneficial effect on diet-induced obesity in mice.

The effect of orally administered commercially available medical cannabis oil on obesity parameters has not been described previously. Evidence suggests that several compounds in the plant may exert anti-obesity effects, including the terpenes D-Limonene and β-Caryophyllene, which are found in Avidekel (Supplementary Table S2) and Erez extracts (Supplementary Table S3), alongside the main phytocannabinoids THC and CBD [19,21,22,23,24]. Whole plant extracts are suggested to be more effective compared to purified isolates administered, an effect that is also known as the ‘entourage effect’ [31].

Our results indicate that THC, which is the main constituent of Erez cannabis extract, exerts anti-obesity effects and prevents weight gain in HFD-fed mice. This could be attributed to the ability of THC to produce tolerance when consumed frequently, as in the case of cannabis users [32]. The tolerance produced by THC leads to reduced CB1 density and coupling efficacy that may be beneficial in obesity, where the endocannabinoid system is overactivated [32]. In support of this, our results show that Erez extract prevented weight gain in mice fed a high-fat diet while not affecting the weights of mice fed a standard diet. Another possible explanation for the resistance to diet-induced obesity, while not affecting caloric intake, is increased energy expenditure induced by THC consumption. Indeed, a recent study showed that daily administration of THC to adolescent mice caused a reduction in weight gain partly due to heightened energy expenditure [33].

HFD-fed mice treated with Erez and Avidekel extract exhibited fewer hypertrophic adipocytes, even though the up-regulation of the pro-inflammatory cytokine il-1β, could be seen in the adipose tissue of both treated groups. Interestingly, adipose tissue inflammation was exacerbated in mice fed a standard diet and treated with medical cannabis extracts, especially that of Avidekel, as demonstrated by the elevated mRNA expression of the macrophage markers F4/80 and CD14 and pro-inflammatory cytokine INF-γ. Because Avidekel extract is high in CBD and contains considerable amounts of the terpenes β-Myrcene and β-caryophyllene (Supplementary Table S4), all of which possess anti-inflammatory properties, it was expected to have an anti-inflammatory effect [23,34,35]. Indeed, a previous study demonstrated the anti-inflammatory properties of Avidekel extract, which overcame the bell-shaped dose–response that is typically seen in treatments with purified CBD [36]. Nevertheless, the majority of publications demonstrated immunomodulatory effects of CBD in pro-inflammatory conditions; however, in basal conditions, CBD can induce a pro-inflammatory response, which is consistent with our observations [37]. It could be speculated that the pro-inflammatory response by the cannabis oil preparations could imply preconditioning of the adipose tissue, enhancing their therapeutic effect [25]. Though this subject is beyond the scope of the study, it is worthy of future research.

Opposing effects of Erez and Avidekel extracts were seen in obesity-induced liver steatosis parameters as the latter demonstrated increased liver weight, lipid droplets, and serum cholesterol levels compared to the former. Overall, Erez extract attenuated liver steatosis, while Avidekel extract increased the severity of liver steatosis as indicated by the low and high steatosis scores. Interestingly, although Avidekel extract and Erez extract contained negligible amounts of THC and CBD, respectively, the liver tissues from both treatments presented considerable amounts of the active molecules and their respective metabolites. Though the large standard deviation can account partially for these results, it is important to consider the implications of the accumulation of THC in certain tissues when consuming CBD-enriched cannabis extracts. The improvement by using Erez extract was associated with decreased levels of the *N*-acylethanolamines (NAEs), PEA and DHEA. Previous studies have shown that the dietary administration of PEA, an endogenous ligand of peroxisome proliferator-activated receptor (PPAR)-α, can improve diet-induced metabolic complications, including liver steatosis [38,39]. Yet, levels of PEA are increased in cirrhotic liver tissues and in activated hepatic stellate cells (HSCs) which play a crucial role in promoting the pathogenesis of liver disease [40,41]. In this regard, the increased levels of PEA in vehicle-treated mice could imply an initial indicator of liver dysfunction, which Erez extract prevented. However, the implication of this finding warrants further investigation. Although DHEA, an ꞷ-3 derived anti-inflammatory lipid mediator, was also significantly decreased by using the Erez extract, the low levels detected in the liver suggest a minor role of this endocannabinoid-like mediator in liver steatosis [42].

Previous studies have attributed the beneficial effect of ECS manipulation in obese settings to changes in the gut microbial community; thus, we aimed to examine changes in the gut microbiota of mice during treatment [6,19]. An alteration in the gut microbiota could be seen initially in mice fed a high-fat diet, as indicated by the lower alpha diversity and increased Firmicutes/Bacteroidetes ratio, both of which are linked to obesity-associated dysbiosis [8]. Interestingly, a negative shift of the gut microbiota, reflected by the decrease in alpha diversity, was observed after the first administration of the Erez extract compared to the Avidekel extract in mice fed a high-fat diet. This initial effect of Erez extract suggests that the treatment has an adverse effect on the gut microbiota, as alpha diversity is considered the most common indicator to assess gut microbiota health [43]. However, this immediate effect did not last, and at the end of the study, no significant alterations could be seen between treatment groups. Moreover, all groups, regardless of treatment, demonstrated a decrease in alpha diversity and an increase in the Firmicutes/Bacteroidetes ratio over time, indicating that time was the dominant factor for gut microbiota alterations.

The current study demonstrates the superiority of THC-enriched cannabis (Erez) extract in preventing HFD-induced weight gain and relieving obesity-induced liver steatosis compared to CBD-rich cannabis extract. In contrast to other publications, our results suggest that the protective effect of Erez extract does not involve changes in the gut microbial community. The improvement in liver steatosis by using Erez extract was concomitant with decreased levels of liver PEA, which could suggest a protective effect on liver function, though this finding could be secondary to the overall effect of Erez extract on obesity.

In summary, our results, jointly with our previous report [24], provide evidence that THC is the main constituent of cannabis able to exert effective anti-obesity properties. The underlying mechanism requires further investigation.

## 4. Materials and Methods

### 4.1. Animals

All procedures were approved by the animal ethics committee of the Hebrew University of Jerusalem (approval number: AG-23-15437-3). Four-week-old C57BL/6 male mice were purchased from Envigo (Rehovot, Israel) and housed in polycarbonate cages under a 12 h light/dark cycle-controlled temperature and constant humidity. Mice were fed ad libitum with either a standard diet (STD; 2018sx; Harlan Teklad) containing 70% carbohydrates, 20% protein, and 10% fat or a high-fat diet (HFD; TD.06414; Harlan Teklad) containing 60% fat, 20% protein, and 20% carbohydrates. The fatty acid profiles of both the STD and HFD are shown in Supplementary Table S1.

### 4.2. Study Compounds

Erez extract (15% THC) and Avidekel extract (30% CBD) were provided by Tikun Olam Ltd. (Tel-Aviv, Israel). Avidekel and Erez oil preparations used in the study were analyzed for phytocannabinoids (Supplementary Figures S1 and S2, respectively) and terpenes (Supplementary Tables S2 and S3, respectively). CoA Terpene profile was provided by Tikun Olam Ltd. (Tel-Aviv, Israel), and phytocannabinoid analysis was performed by the laboratory for the Mass Spectrometry and Chromatography, the Hebrew University, Rehovot, Israel. Briefly, quantitative analyses of phytocannabinoids in plants and cannabis preparations were carried out using the Dionex Ultimate 3000 RS HPLC, which consisted of a quaternary pump, autosampler, column department, and diode-array detector. Analytes were separated on Acclaim C18 column (250 × 3 mm, 3 µm, Dionex, Sunnyvale, CA, USA) using acetonitrile/water with 0.1% acetic acid gradient. Standards of phytocannabinoids used for identification and quantitative analysis were purchased from Restek (Bellefonte, PA, USA).

### 4.3. Treatments

Mice were fed with HFD or STD for 14 weeks and then randomly divided into treatment groups (*n* = 5–7). The dosing schedule was based on consideration of “frequent cannabis use” as determined elsewhere [44]. Mice were administered 10 µL of THC, CBD, or vehicle control (olive oil) three times a week via noninvasive oral delivery using a micropipette. We incorporated a ramping-dose method of the respective active molecule (THC or CBD), from 10 mg/kg (5 weeks) to 30 mg/kg (5 weeks), to account for the induction of tolerance, which is known to occur rapidly with prolonged cannabis use [32]. Weight and food intake were measured twice a week during the treatment regime. At the end of the treatments, mice were euthanized via isoflurane overdose, their blood was collected, and serum cholesterol was quantified using a Cobas C-111 chemistry analyzer (Roche Diagnostics, Rotkreuz, Switzerland). Liver and adipose tissues were also collected and further analyzed.

### 4.4. DNA Extraction and Sequencing from Mouse Feces

Feces were collected at various time points and kept frozen at −20 °C. DNA was extracted by 0.1 mm glass beads disruption in the presence of Tris-saturated phenol, followed by phenol–chloroform extraction and isopropanol precipitation. 16S rDNA library was prepared using V4 primers 515F (GTGYCAGCMGCCGCGGTAA) and 806R (GGACTACNVGGGTWTCTAAT), according to the Earth Microbiome Project protocol [45]. Samples were sequenced by Hylabs (Rehovot, Israel) on an Illumina Miseq platform using a V2 250 bp paired-end reagent kit. Sequence processing and taxonomy assignment were performed using QIIME2. Amplicon sequence variants (ASVs) were determined with the Dada2 plugin (version 2020.11.1) using the denoise-paired method. All reads were truncated at position 200; otherwise, default parameters were used. Taxonomy was assigned using a naive Bayes classifier trained on the Greengenes database [46]. All samples were normalized to 6000 reads per sample, and ASVs with under 5 reads were discarded.

### 4.5. Liver Triglyceride Quantification

Briefly, 80 mg of liver tissue was homogenized in 0.5 mL of ice-cold 1:1 methanol–Tris (50 mM Tris buffer pH 8) solution. The homogenate was washed twice with 1 mL ice-cold chloroform–methanol (1:1 *v*/*v*), and 0.5 mL of 50 mM Tris pH was added and vortexed, and then the mixture was centrifuged at 3000× *g* at 4 °C for 10 min. The organic phase was transferred to a glass tube and underwent two more extractions with ice-cold chloroform. Then, 1 mL of 5% Triton X-100 in chloroform was added and mixed using vortex. Chloroform was evaporated with N2 gas at 32 °C, and triglycerides were reconstituted with 1 mL of ddH2O. The extracted triglycerides were measured using a colorimetric kit according to manufacturer instructions (Cayman Chemicals, Ann Arbor, MI, USA).

### 4.6. Histology and Microscopy

Adipose and liver tissues were embedded in paraffin and sectioned to 5 µM thickness on superfrost slides. Slides were stained using hematoxylin (Hebrew University pathology core facility). The stained slides were photographed using a Canon microscope with a mounted Olympus camera. Adipose cell size and hepatic lipid droplet counts were calculated using Fiji software (version 1.53) [47].

### 4.7. Liquid Chromatography–High-Resolution Mass Spectrometry (LC–HRMS) Chemical Analysis

The analysis of endocannabinoids, endocannabinoid-like lipids, and pure THC and CBD from liver samples was performed using Thermo Scientific ultra HPLC system coupled with a Q Exactive^TM^ Focus Hybrid Quadrupole-Orbitrap MS, according to a method developed and validated by David Meiri and colleagues [48].

### 4.8. RNA Extraction and Real-Time Quantitative PCR

RNA was extracted from the hepatic tissues using the NucleoSpin RNA extraction kit (Mecherey-Nagel, GmbH, Düren, Germany) according to the manufacturer’s instructions. Additionally, RNA was extracted from the adipose tissue using RNeasy Lipid Tissue Mini Kit (Qiagen, Hildan, Germany) according to the manufacturer’s instructions. Briefly, 1 µg of total RNA was used to create cDNA using the qScript cDNA Synthesis Kit (Quantabio, Beverly, MA, USA). Relative gene expression was analyzed on QuantStudio 1 Real-Time PCR using SYBR^®^ Green-Based qPCR (Thermo Fisher, Waltham, MA, USA). Real-time PCR primers are listed in Supplementary Table S4.

### 4.9. Statistics

Statistical analyses were performed using GraphPad Prism 9 (GraphPad software, version 9.0.0). Unless otherwise stated, data are expressed as mean ± SEM. Normally distributed data, based on examination of Shapiro–Wilk testing, were tested using two-way ANOVA, followed by Tukey’s post hoc test. Alpha diversity metrics were analyzed using Mann–Whitney for two group comparisons, and the Kruskal–Wallis test was used for multiple groups. A difference of *p* < 0.05 was considered statistically significant.

## Figures and Tables

**Table 1:** Levels of endocannabinoids in liver.

	Lipid Family	ECs (ng/g Tissue)	Vehicle—HFD	Avidekel—HFD	Erez—HFD
Liver	NAEs	AEA	23.8 ± 4.8	22.9 ± 3.9	15.8 ± 2.9
		DHEA	6 ± 1.5 ^a^	5.1 ± 0.9 ^ab^	3.3 ± 0.2 ^b^
		LEA	36.8 ± 7.8	23.3 ± 4.7	23.5 ± 3.8
		OEA	33 ± 2.4	35 ± 3.8	21 ± 3.1
		PEA	157 ± 16 ^a^	126 ± 17 ^ab^	91 ± 13 ^b^
		SEA	134 ± 27	117 ± 24	85 ± 13
	2-MAGs	2-AG	1764 ± 572	526 ± 64	691 ± 37
		2-LG	10,362 ± 4853	2362 ± 387	4023 ± 759
	NAAs	NL-Gly	122 ± 41	101 ± 17	70 ± 13
		NP-Gly	366 ± 70	393 ± 47	206 ± 35
		NA-Gly	117 ± 44	115 ± 21	98 ± 23
		NDH-Gly	106 ± 33	77 ± 16	58 ± 10
		NA-Ser	46 ± 19	37 ± 8.9	27 ± 6.9
		NA-GABA	6 ± 2.5	6 ± 1.6	2.6 ± 0.3
		NA-Ala	91 ± 32	88 ± 15	69 ± 16

Data are shown as mean ± SEM with *n* = 3–4 animals per group. Statistical significance was determined using the non-parametric Kruskal–Wallis test. Different subscribed letters indicate significant differences between groups, *p* < 0.05; 2-AG, 2-arachidonoyl glycerol; AEA, arachidonoyl ethanolamide; DHEA, docosahexanoyl ethanolamide; LEA, linoleoyl ethanolamide; OEA, oleoyl ethanolamide; PEA, palmitoyl ethanolamide; SEA, stearoyl ethanolamide; DEA, docosatetraenoyl ethanolamide; 2-LG, linoleoyl glycerol; NL-Gly, N-linoleoyl glycine; NP-Gly, N-palmitoyl glycine; NA-Gly, N-arachidonoyl glycine; NDH-Gly, N-docosahexaenoyl glycine; NA-Ser, N-arachidonoyl serine; NA-GABA, N-arachidonoyl gamma-aminobutyric acid; NA-Ala, N-arachidonoyl alanine.

## Data Availability

The data underlying this article will be shared upon reasonable request by the corresponding author.

## Supplementary Materials

The following supporting information can be downloaded at: https://www.mdpi.com/article/10.3390/ijms25021073/s1.
