# Supplementary material for: The Effect of Oil-Based Cannabis Extracts on Metabolic Parameters and Microbiota Composition of Mice Fed a Standard and a High-Fat Diet
Source: Int J Mol Sci. 2024 Jan 16;25(2):1073. doi: 10.3390/ijms25021073 (PMC10816190; doi:10.3390/ijms25021073)
Supplement: Supplementary file 1 [file ijms-25-01073-s001.zip › ijms-2793602-supplementary.pdf]

Supplementary Information

The effect of commercially available medical cannabis oils on obesity parameters and microbiota composition

Adi Eitan, Ofer Gover, Liron Sulimani, David Meiri, Naama Shterzer , Erez Mills, and Betty Schwartz

Table S1: Fatty acid composition of the STD and HFD used in this study

	HFD	STD
Total fat (g/kg)	342.7	62
Saturated fat (g/kg)	119.2	0.63
C14:0 myristic	3.1	-
C16:0 Palmitic	74.6	0.49
C18:0 Stearic	41.5	0.14
Monounsaturated fat (g/kg)	134	9.1
C16:1 Palmitoleic	6.2	-
C18:1 Oleic	127.8	8.4
Polyunsaturated fat (g/kg)	77.2	23.8
C18:2 Linoleic	71.7	21.7
C18:3 Linolenic	5.5	0.21
ω6:ω3 ration	13	10

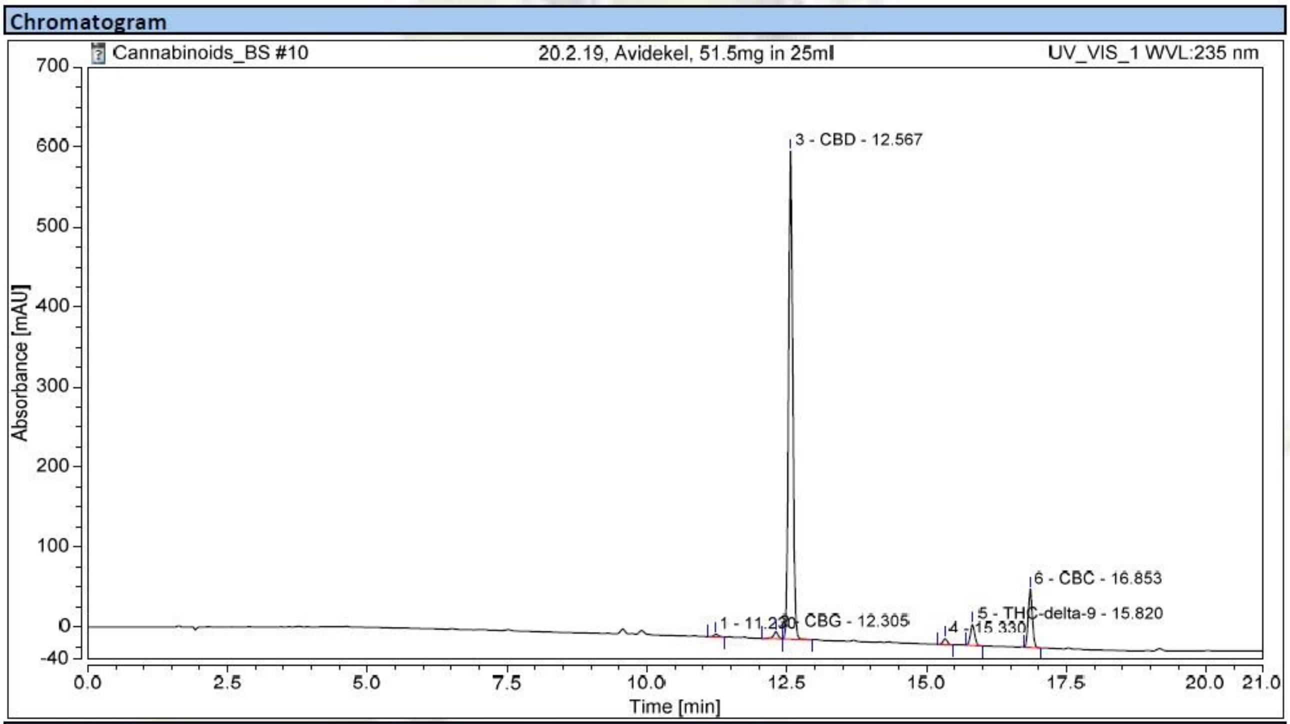

Integration Results

No.	Peak Name	Retention Time min	Area mAU*min	Height mAU	Relative Area %	Relative Height %	Amount weight %
1		11.230	0.348	3.317	0.55	0.46	n.a.
n.a.	CBDA	n.a.	n.a.	n.a.	n.a.	n.a.	n.a.
2	CBG	12.305	0.833	9.053	1.33	1.24	0.47
3	CBD	12.567	52.583	609.957	83.76	83.71	29.19
n.a.	CBN	n.a.	n.a.	n.a.	n.a.	n.a.	n.a.
4		15.330	0.659	7.546	1.05	1.04	n.a.
5	THC-delta-9	15.820	2.264	26.038	3.61	3.57	1.17
n.a.	THC-delta-8	n.a.	n.a.	n.a.	n.a.	n.a.	n.a.
6	CBC	16.853	6.094	72.756	9.71	9.98	1.16
n.a.	THCA	n.a.	n.a.	n.a.	n.a.	n.a.	n.a.
**Total:**			**62.781**	**728.668**	**100.00**	**100.00**	

Figure S1. Chromatogram of Avidekel extract. Phytocannabinoid profiling of Avidekel oil extract was performed according to the method described in the materials and method section.

Chromatogram

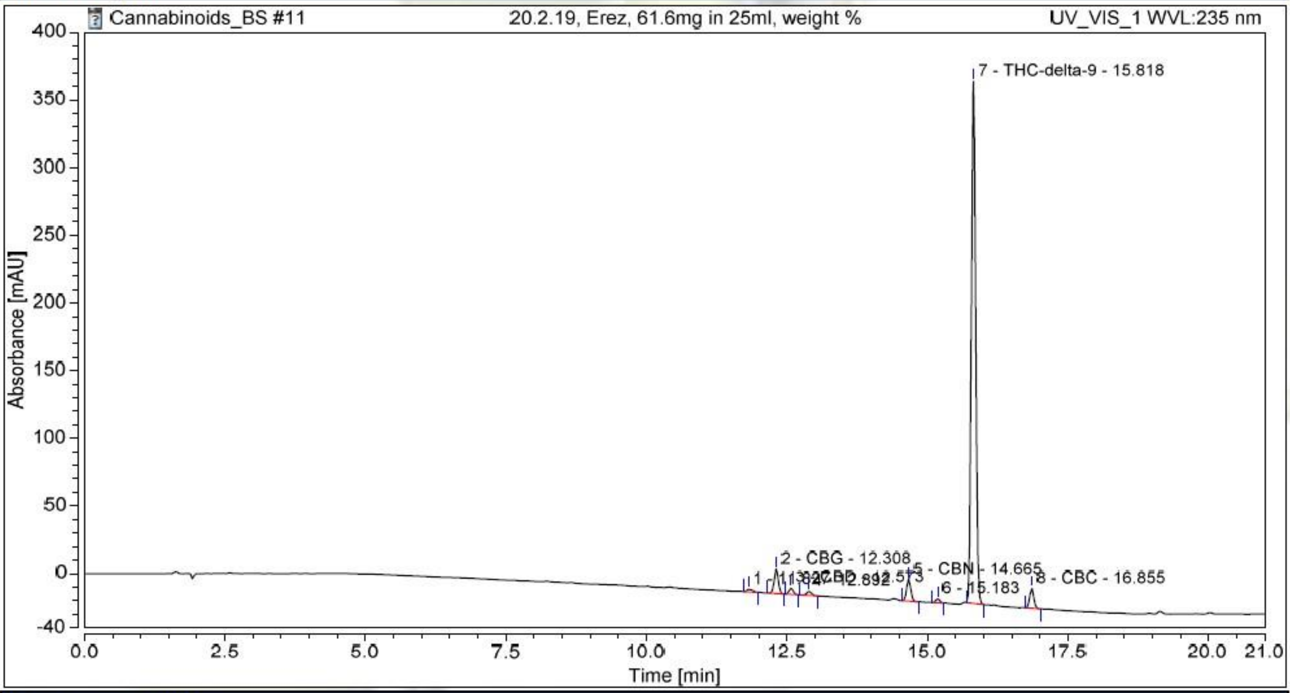

Integration Results

No.	Peak Name	Retention Time min	Area mAU*min	Height mAU	Relative Area %	Relative Height %	Amount weight %
n.a.	CBDA	n.a.	n.a.	n.a.	n.a.	n.a.	n.a.
1		11.827	0.224	2.007	0.57	0.45	n.a.
2	CBG	12.308	1.514	18.165	3.86	4.06	0.71
3	CBD	12.573	0.392	4.696	1.00	1.05	0.18
4		12.892	0.297	3.130	0.76	0.70	n.a.
5	CBN	14.665	1.369	15.914	3.49	3.56	0.27
6		15.183	0.238	2.896	0.61	0.65	n.a.
7	THC-delta-9	15.818	33.968	385.683	86.56	86.23	14.81
n.a.	THC-delta-8	n.a.	n.a.	n.a.	n.a.	n.a.	n.a.
8	CBC	16.855	1.238	14.804	3.15	3.31	0.20
n.a.	THCA	n.a.	n.a.	n.a.	n.a.	n.a.	n.a.
**Total:**			**39.240**	**447.295**	**100.00**	**100.00**	

Figure S2. Chromatogram of Erez extract. Phytocannabinoid profiling of Erez oil extract was performed according to the method described in the materials and method section.

Table S2: Terpenes assayed by GC-FID - Avidekel extract

Test	mg/g	% Total Terpenes
**Terpenes Assay By GC-FID**		
a-pinene	0.02	0.3
Camphene	0.00	0.0
Myrcene	1.98	28.4
b-Pinene	0.05	0.7
d-Limonene	0.90	12.9
b-ocimene	0.17	2.4
Eucalyptol	0.32	4.6
y-Terpinene	0.06	0.9
Terpinolene	0.19	2.8
Linalool	1.24	17.8
Geraniol	0.00	0.0
b-Caryofhyllene	1.67	24.0
a-Humulene	0.37	5.3
Nerolidol	0.00	0.0
a-Bisabolol	0.00	0.0
**Total**	6.97	100

Table S3: Terpenes assayed by GC-FID - Erez extract

Test	mg/g	% Total Terpenes
**Terpenes Assay By GC-FID**		
a-pinene	0.01	0.2
Camphene	0.00	0.0
Myrcene	0.13	3.0
b-Pinene	0.03	0.6
d-Limonene	0.12	2.8
b-ocimene	0.12	2.6
Eucalyptol	0.00	0.0
y-Terpinene	0.04	0.9
Terpinolene	0.17	3.9
Linalool	0.56	12.5
Geraniol	0.00	0.0
b-Caryofhyllene	2.51	56.3
a-Humulene	0.77	17.2
Nerolidol	0.00	0.0
a-Bisabolol	0.00	0.0
**Total**	4.46	100

Table S4: Primer list

Gene	Forward primer / Reverse primer	Accession number
	**Reverse primer**	
F4/80	CCTTTGGCTATGGGCTTCCA	NM_001355722.1
	CCTCAGAACCCACAGTGTCC	
CD14	CTGAAGCCTTTCTCGGAGCC	NM_009841.4
	GCATAAGCTTCATGGTCGGTAGA	
INF-γ	AGCGCCAAGCATTCAATGAG	NM_ 008337.4
	AATCTCTTCCCCACCCCGAA	
IL-1β	TCGCTCAGGGTCACAAGAAA	NM_ 008361.4
	CATCAGAGGCAAGGAGGAAAAC	
